# Effectiveness and Economic Evaluation of Chiropractic Care for the Treatment of Low Back Pain: A Systematic Review of Pragmatic Studies

**DOI:** 10.1371/journal.pone.0160037

**Published:** 2016-08-03

**Authors:** Marc-André Blanchette, Mette Jensen Stochkendahl, Roxane Borges Da Silva, Jill Boruff, Pamela Harrison, André Bussières

**Affiliations:** 1 Public Health PhD Program, School of Public Health, University of Montreal, Montreal, QC, Canada; 2 Nordic Institute of Chiropractic and Clinical Biomechanics, Odense, Denmark; 3 Faculty of Nursing, University of Montreal, Montreal, QC, Canada; 4 School of Physical and Occupational Therapy, Faculty of Medicine, McGill University, Montreal, QC, Canada; 5 Centre de Recherche Interdisciplinaire en Réadaptation de Montréal, Montreal, QC, Canada; 6 Département chiropratique, Université du Québec à Trois-Rivières, Trois-Rivières, QC, Canada; University Medical Center Göttingen, GERMANY

## Abstract

**Background Context:**

Low back pain (LBP) is one of the leading causes of disability worldwide and among the most common reasons for seeking primary sector care. Chiropractors, physical therapists and general practitioners are among those providers that treat LBP patients, but there is only limited evidence regarding the effectiveness and economic evaluation of care offered by these provider groups.

**Purpose:**

To estimate the clinical effectiveness and to systematically review the literature of full economic evaluation of chiropractic care compared to other commonly used care approaches among adult patients with non-specific LBP.

**Study Design:**

Systematic reviews of interventions and economic evaluations.

**Methods:**

A comprehensive search strategy was conducted to identify 1) pragmatic randomized controlled trials (RCTs) and/or 2) full economic evaluations of chiropractic care for low back pain compared to standard care delivered by other healthcare providers. Studies published between 1990 and 4^th^ June 2015 were considered. Primary outcomes included pain, functional status and global improvement. Study selection, critical quality appraisal and data extraction were conducted by two independent reviewers. Data from RCTs with low risk of bias were included in a meta-analysis to determine effect estimates. Cost estimates of full economic evaluations were converted to 2015 USD and results summarized using Slavin’s qualitative best-evidence synthesis.

**Results:**

Six RCTs and three full economic evaluations were scientifically admissible. Five RCTs with low risk of bias compared chiropractic care to exercise therapy (n = 1), physical therapy (n = 3) and medical care (n = 1). Overall, we found similar effects for chiropractic care and the other types of care and no reports of serious adverse events. Three low to high quality full economic evaluations studies (one cost-effectiveness, one cost-minimization and one cost-benefit) compared chiropractic to medical care. Given the divergent conclusions (favours chiropractic, favours medical care, equivalent options), mixed-evidence was found for economic evaluations of chiropractic care compared to medical care.

**Conclusion:**

Moderate evidence suggests that chiropractic care for LBP appears to be equally effective as physical therapy. Limited evidence suggests the same conclusion when chiropractic care is compared to exercise therapy and medical care although no firm conclusion can be reached at this time. No serious adverse events were reported for any type of care. Our review was also unable to clarify whether chiropractic or medical care is more cost-effective. Given the limited available evidence, the decision to seek or to refer patients for chiropractic care should be based on patient preference and values. Future studies are likely to have an important impact on our estimates as these were based on only a few admissible studies.

## Background

Low back pain (LBP) is the most common occupational disorder in North America [1, 2], a major cause of work absenteeism [3, 4] and a leading cause of disability worldwide [5]. The 2010 Global Burden of Disease Study revealed that the LBP disability-adjusted life years increased from 58.2 million in 1990 to 83.0 million in 2010[6], although the majority of LBP patients experience non-specific symptoms that cannot be attributed to a serious disease [7].The global point prevalence of LBP is 9.4% [6] and the life time prevalence is around 85% [8, 9].

A number of factors drive patients’ choice for a specific health provider. First, access to professionals is influenced by a traditional medical model of referral [10]. Other structural factors include the regional supply of providers [11, 12] or coverage of provider services in medical insurance schemes [13]. Financial reasons (such as out of pocket expenses), socio-cultural (such as traditions) and personal beliefs and preferences are also driving factors [14]. Perhaps as a result, patients with LBP tend to first consult general physicians [10]. However, an increasing number of LBP sufferers seek care directly from other healthcare professionals [15, 16]. From 2006 to 2010, the proportion of patients self-referring to physiotherapists in The Netherlands rose from 22% to 43%[17]. LBP patients also commonly seek chiropractic care [18–20]. At least one third of back pain patients in Denmark choose to see a chiropractor as their entry into the healthcare system [15].

North American health technology assessments on chiropractic care conducted over a decade ago were unable to provide clear guidance to inform decision-making on the effectiveness and cost effectiveness of chiropractic care compared to medical and physiotherapy care [21, 22]. This was primarily because their analysis was based on a limited number of studies of acceptable methodological quality and partial economic evaluations (cost description, cost analysis and cost-outcome description) [21, 22].

In a fastidious trial, the efficacy of spinal manipulative therapy (SMT) is tested under optimal conditions in order to isolate its effect from the confounding factors [23]. While in a pragmatic trial, the effectiveness of chiropractic care is tested in close to ‘real-world’ clinical settings to measure the degree of beneficial effect under this type of conditions [23]. More recently, systematic reviews on the effectiveness of SMT combined the two types of studies and concluded that this approach was as effective as other commonly used treatment modalities [24–26]. While SMT is an important component of chiropractic care [27], these providers commonly use multimodal care to treat their patients with LBP in order to enhance treatment outcome [28] [29]. SMT is also performed by a range of other healthcare professionals [25, 26]. Thus, studies evaluating the effectiveness of SMT can guide clinicians in the choice of treatment modality, but provide little guidance to patients regarding which healthcare provider they should seek care from.

When referring to other providers, inter-professional relations appear to be important to clinicians, where cost is likely a major driver for third party payers when deciding to include a type of provider within a healthcare plan [30–32]. However, healthcare providers, policy makers and third party payers should likely consider the clinical effectiveness, the cost-effectiveness, the safety of the approach, and patient preference and values when referring (LBP) patients for a specific type of care or including a service in a medical insurance scheme, [24, 25, 33]. Full economic evaluation (cost-effectiveness analysis, cost-utility analysis, cost-benefit analysis) of standard care practice offers the advantage of simultaneously considering the resources involved (costs) and the health outcomes (outputs) [34]. This is important when formulating recommendations on the optimal use of healthcare resources [35]. This review therefore aimed to synthetize recent high quality evidence to better inform patients, clinicians, policy makers, and third party payers about the clinical effectiveness and cost-effectiveness of standard chiropractic care for LBP in comparison to usual standard care provided by other healthcare providers. The specific objectives of this review were: 1) to estimate the extent to which chiropractic care is effective for adult patients with non-specific low back pain compared to other conservative care approaches (e.g. medical care and physiotherapy), and 2) to systematically review the literature of full economic evaluation of chiropractic care for adult patients with non-specific LBP compared with other conservative care approaches conducted from any perspective.

## Materials and Methods

### Eligibility criteria

We conducted two systematic reviews: 1) a review of clinical effectiveness and 2) an economic review.

#### Study characteristics

For the clinical effectiveness review, only randomised controlled trials were eligible for inclusion. For the economic review, studies with a full economic evaluation (i.e. cost-effectiveness, cost-utility, cost-benefit analyses and cost-minimization analysis alongside a clinical trial [36]) were eligible.

#### Population under study

Adult patients (≥ 18 years) with non-specific LBP with or without sciatica of any duration were eligible for inclusion. We excluded studies reporting on spinal pain without separate results for LBP and studies examining specific pathologies (e.g., disc herniation or compression fractures).

#### Types of interventions

We compared pragmatic trials of chiropractic standard care with standard care delivered by other healthcare providers. Chiropractic standard care was defined as patient-centred, multimodal care (e.g. combinations of SMT, soft tissue techniques, prescription of exercise, advice and reassurance) planned and delivered by a licensed chiropractor. We excluded studies investigating chiropractic care combined with care delivered by other healthcare providers, studies investigating a specific treatment modality or technique, and trials aiming to isolate the effect of SMT (i.e. fastidious trials). The lead authors of potentially relevant studies were contacted for further clarity regarding type of care.

The comparators were conservative, standard care for LBP delivered by other healthcare providers (e.g. medical doctor, physical therapist, exercise therapist or acupuncturist). Studies were judged eligible if the original author, when contacted for clarification, considered the comparator group as standard care. Study arms including surgical treatment or multidisciplinary care were excluded.

### Types of outcome measures

- For the clinical effectiveness review:

Primary outcomes:

- Pain (e.g., visual analogue scale, numerical rating scale, McGill pain score)
- Functional status (e.g. Roland-Morris questionnaire, Oswestry Disability Index)
- Global improvement (e.g., the proportion of patients recovered)

Secondary outcomes:

- Health related quality of life (e.g., SF-36, EuroQol)
- Return to work (e.g. number of days to return to work or proportion of patients at work)
- Adverse events

- For the economic review: an incremental measure of the extra cost required to improve an additional unit of outcome (e.g., an incremental cost-effectiveness ratio (ICER) or an incremental net benefit measure) with the exception of cost-minimization studies for which only costs were considered.

#### Additional criteria

Studies published in languages other than English or French, duplicate publications and studies without full text manuscript available (e.g. abstracts, conference proceedings, presentations) were excluded.

### Information sources

#### Electronic searches

We developed our search strategies with an experienced health sciences librarian (JB) (The complete search strategies can be found in the published protocol[37]). A second librarian (PH) reviewed the search strategy for completeness and accuracy. We searched the following databases: Ovid Medline, Ovid AMED, Ovid EMBASE, CINAHL, the Cochrane Database of Systematic Reviews, and PubMed. We further searched for economic evaluations in four additional databases: Index to Chiropractic Literature (ICL); Cochrane Library; Health Technology Assessment Database; and ECONLIT. We searched all bibliographic databases from 1990 to 7th June 2016.

The search strategies (clinical effectiveness and economic evaluation) were first developed in MEDLINE and subsequently adapted to the other bibliographic databases. The search terms included subject headings (eg, MeSH) specific to each database and free-text words relevant to low back pain. We used Endnote (version X7.3.1, Thomson Reuters, Philadelphia, PA, USA) to create a bibliographic database to manage the search results.

#### Other resources

We screened reference lists of relevant publications, including reviews and meta-analyses, for relevant articles, and reviewed the gray literature available from the following websites: Canadian Institute for Health Information (CIHI); Canadian Agency for Drugs and Technologies in Health (CADTH); Canadian Institute of Health Research (CIHR); Tufts Medical Center Cost-effectiveness Analysis Registry; Agency for Healthcare Research and Quality; National Institute for Health Research Health Technology Assessment program; and National Institute for Health and Care Excellence (NICE).

### Study selection

Two pairs of authors independently screened titles, abstracts, and full text papers for the clinical effectiveness (MAB and MJS) and for the economic evaluation reviews (MAB and AB). A third reviewer (MJS) was included in the full text screening of the economic evaluations. Reviewers met to resolve disagreements and reach consensus on the eligibility of studies.

### Quality assessment

#### Clinical effectiveness studies

Two reviewers with expertise related to chiropractic care and clinical trials (MAB and MJS) critically appraised the internal validity of included studies using the 13 criteria recommended by the Cochrane Back Review Group [38]. Studies that met at least 6 criteria out of 13 were considered low risk of bias. Items related to the blinding of patients, care providers, and outcome assessors (patient reported outcomes) were reported in the risk of bias assessment, but cannot be fulfilled when studying the outcome of different groups of healthcare providers.

#### Economic evaluations

The methodological quality of the economic evaluation was independently assessed by two reviewers using the Drummond BMJ Check list [39–41]: one with expertise related to chiropractic care and epidemiology (MAB) and one with expertise related to healthcare administration and health economics (RBDS). The checklist included 35 items related to study design; data collection; data analysis; and interpretation of results. Additionally, the reviewers formulated a qualitative appreciation of the quality level (low, medium, high) of every study:

**High quality**: The majority of the quality assessment criteria are met. There is little or no methodological flaw that might influence the study conclusion.

**Medium quality**: Most of the quality assessment criteria are met. Some flaws in the study may influence the study conclusion.

**Low quality**: Either most of the quality assessment criteria are not met, or significant flaws relating to key aspects of study are likely to influence the study conclusion.

### Data extraction

Data extraction was performed by the lead investigator (MAB) and checked for accuracy against the original publication by a second reviewer for the clinical effectiveness trial (MJS) and the economic evaluation (RBDS).

The standard form developed by the Cochrane back review group [42] was used to extract descriptive and outcome data from the clinical effectiveness studies. Authors of studies only reporting between group differences [43–46] were unsuccessfully contacted in order to obtain mean and standard deviation of primary outcomes at relevant time points. Therefore, the means were inferred from graphical representations and our analysis were based on the reported between group differences. Data extracted from studies at high risk of bias were not reported.

A customized data extraction form [37] was used for the economic evaluations. Authors of the economic evaluations were contacted in order to gain precision about the perspective of analysis and the type of care considered.

### Data analysis

#### Clinical effectiveness studies

Primary and secondary outcomes of studies with low risk of bias were evaluated in meta-analyses. Final scores of reported outcomes were used for the meta-analyses. Data were estimated, using the Review Manager calculator (RevMan version 5.3, The Cochrane Collaboration, Copenhagen, Denmark) when change scores and between group differences were reported. Outcomes were assessed at 1, 3, and 12 months. In order to minimize clinical diversity, we stratified by: healthcare provider (chiropractic care versus medical care, physical therapy and exercise therapy), symptom duration (sub-acute/chronic (6 weeks or more) and mix/not specified), and outcomes (type of outcome and time of assessment). To facilitate comparison between different instruments, comparisons were made using standardized mean difference for continuous outcomes and a risk ratio for dichotomous outcomes. Due to substantial heterogeneity, we used a random effect model. Heterogeneity was investigated by subjective interpretation and by statistical testing using the Q (Chi^2^), Tau^2^ and I^2^ test. We, *a priori*, determined a cut-off of 40% at the I^2^ test for reporting pooled estimates. However, due to the limited number of studies, we decided to present the pooled results along with potential sources of heterogeneity even if this criterion was not met. Sensitivity analysis by adding studies with high risk of bias and construction of funnel plots to evaluate possible publication bias were planned [37], but not conducted because the high risk of bias study retrieved only reported results graphically and the number of studies retrieves was too low to construct a meaningful funnel plot. All meta-analyses calculations and related metrics were conducted in Review Manager for Mac OS X (RevMan version 5.3, The Cochrane Collaboration, Copenhagen, Denmark).

#### Economic evaluations

In order to compare costs across different currencies and account for differential timing, costs of the original study were converted to 2015 United-States (US) dollar using a web-based tool based on purchasing power parities (PPP) and gross domestic product deflators (GDPD) values from the World Economic Outlook Database [47]. The level of evidence of the economic findings was assessed using a 5-point ordinal scale defined by Slavin’s qualitative best-evidence synthesis approach [48]. The following criteria were assessed in stepwise descending manner:

**Strong Evidence**: minimum of 3 high quality studies; at least three-quarters of high and medium quality studies must concur on findings.

**Moderate Evidence**: minimum of 2 high quality studies, or 3 of medium and high quality; more than two thirds of all studies must report consistent findings.

**Limited Evidence**: minimum of 1 high quality study or 2 medium quality studies, more than 50% of all studies must report consistent findings.

**Mixed Evidence**: findings from medium and high quality studies are contradictory.

**Insufficient/No Evidence**: no high quality studies; one or no medium quality studies; any number of low quality studies.

### Reporting

The systematic review was organized and reported based on the Preferred Reporting Items for Systematic Reviews and Meta-Analyses statement[49].

### Systematic review registration

The protocol was registered with the International Prospective Register of Systematic Reviews on October 20, 2014 (CRD42014008746) at: http://www.crd.york.ac.uk/PROSPERO.

FDA device/drug status: Not applicable.

## Results

### Clinical effectiveness

#### Study selection

**Fig 1:**
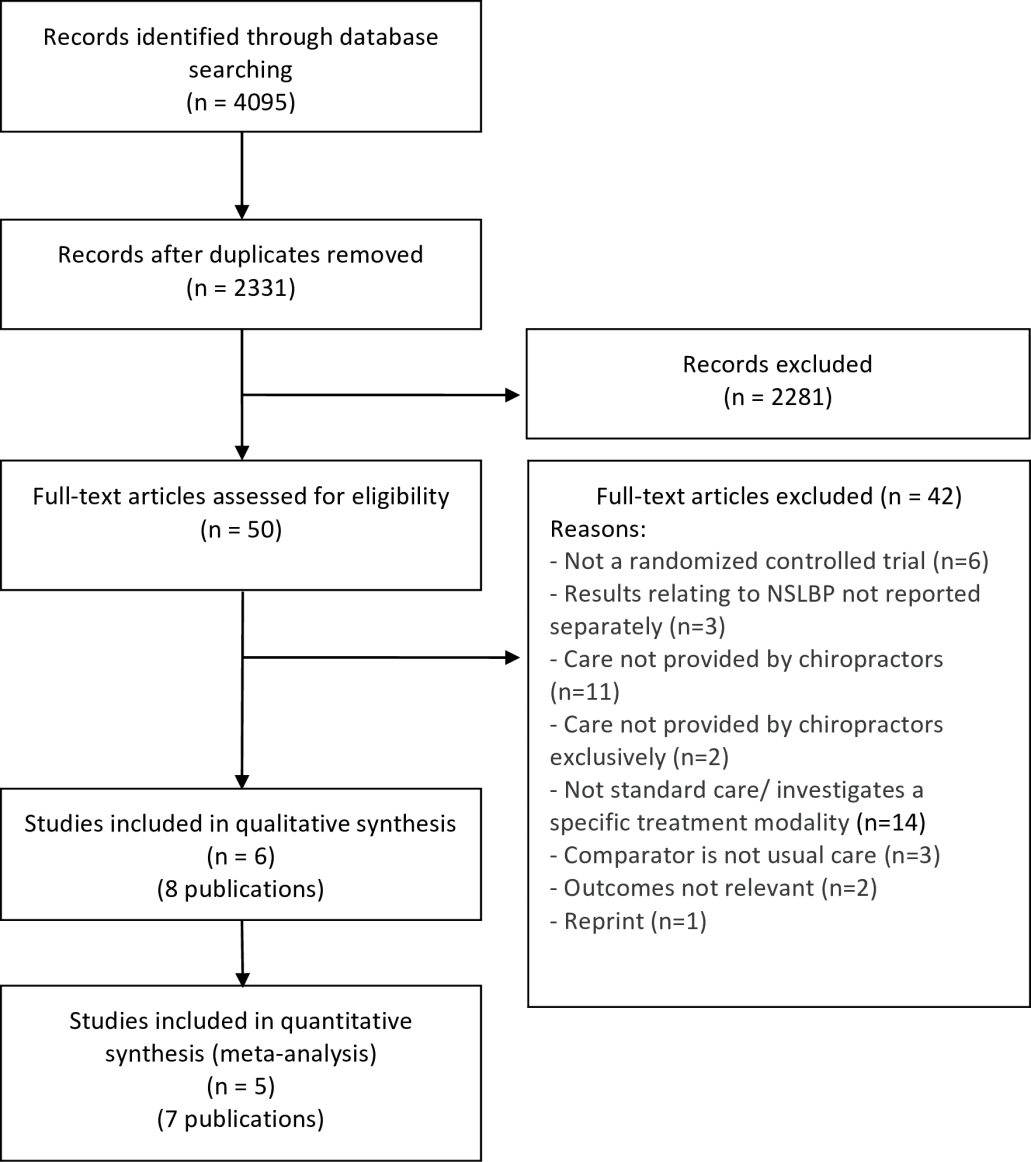
Flow diagram for the selection of clinical effectiveness studies.

Our search retrieved 4,095 articles. We removed 1,764 duplicates and screened 2,331 articles for eligibility (Fig 1). After screening, 2,281 articles did not meet our selection criteria, whereas 50 citations were assessed for eligibility in full-text. Eight citations [43–46, 50–53] originating from six individual studies were included in the review (Fig 1). The main reasons for exclusion after full test screening were: care not provided by a chiropractor or investigation of a specific treatment modality. One potentially relevant study with no results available was identified on clinicaltrials.gov (NCT01357343).

#### Study characteristics

Characteristics of the included studies are presented in Table 1. One Canadian study [52] comparing chiropractic care to physical therapy care had a high risk of bias; therefore we did not include its results into our quantitative analysis and did not further report study findings. The remaining five low risk of bias studies were published between 1990 and 2011 and were conducted in the US (n = 3) [45, 46, 50–52], United Kingdom (UK, n = 1) [43, 44], and Denmark (n = 1) [53]. Chiropractic care was compared to exercise therapy care in one study [50], to physical therapy care in four studies [43, 44, 51, 53] and to medical care in one study[45, 46]. Cohort sizes varied from 155–741 participants and follow-up periods varied from one to three years. We obtained effect estimates for all our primary and secondary outcomes with the exception of return to work. Only the two most recent studies reported responder analysis [50, 53].

**Table 1:** Characteristics of the clinical effectiveness studies included into the quantitative synthesis of Chiropractic care for non-specific low back pain.

First Author, Year, Country and Setting	Participants and Indication	Comparative Treatments	Follow-up assessment	Relevant outcomes
- Bronfort 2011[50] - USA, University research clinic in Bloomington, MN	18 to 65 years old with mechanical LBP of at least 6-week duration with or without radiating pain. (sub-acute/chronic)	• Chiropractic care once to twice per week for 15 to 30 minutes including: SMT and few minutes of soft-tissue massage, ice, or heat (n = 100). • Supervised exercise therapy provided by exercise therapists (n = 100).	• Total: 1 year • Relevant for this review: 1 month (week 4), 3 months (week 12), 12 months (week 52)	- Functional status (Roland-Morris 0–23) - Health related quality of life (SF-36 physical and mental scales) - Global improvement (1 = complete improvement, 9 = twice as bad) - Adverse events
- Cherkin 1998 [51] - USA, Group Health Cooperative of Puget Sound (HMO), Seattle, WA	Patients 20 to 64 years of age who saw their primary care physician for low back pain and who still had pain seven days later. (mix/not specified)	• Chiropractic care: according to usual clinicians procedures including recommendations about exercise and activity restrictions (n = 122). • Physical therapy care: provided by therapists trained by the McKenzie Institute faculty. Subjects received McKenzie’s Treat Your Own Back book and a lumbar-support cushion. Therapists were instructed to avoid therapies such as heat, ice, transcutaneous electrical nerve stimulation, ultrasonography, and back classes (n = 133).	• Total: 2 years • Relevant for this review: 1 month (week 4), 3 months (week 12)*	- Functional status (Roland-Morris 0–24) - Adverse events
- Herzog 1991 [52] - Canada, Unknown setting in Calgary, Alberta	Ambulatory patient between 18 and 50 years old with a sacroiliac joint problem since at least one month. (sub-acute/chronic)	• Chiropractic care: SMT and the optimal treatment modality to the discretion of the chiropractor** for 10 sessions over 4 week (n = 16). • Physical therapy care: back school therapy for 10 sessions over 4 week (n = 13).	• Total: 1 month (week 4, treatment completion) • Relevant for this review: none	- Actual pain (VAS 0–10) - Functional status (Oswestry 0–100)
- Hurwitz 2002 [45, 46] - USA, 3 Primary Care Centers of a 100 000 member health-care network based in southern California	HMO member of at least 18 years old with a complaint of low back pain with or without leg pain. (mix/not specified)	• Chiropractic Care: SMT, instruction in strengthening and flexibility exercises, and instruction in proper back care (n = 169). • Medical care: One or more of the following: instruction in proper back care and strengthening and flexibility exercises; prescriptions for pain killers, muscle relaxants, anti-inflammatory agents, and other medications used to reduce or eliminate pain or discomfort; and recommendations regarding bed rest, weight loss, and physical activities (n = 170).	• Total: 1.5 year • Relevant for this review: 1 month (week 6), 12 months	- Average pain (VAS 0–10) - Functional status (Roland-Morris 0–24)Adverse events
- Meade 1990 [43, 44] - United-Kingdom, 11 centres with hospital and chiropractic clinics within a reasonable distance	Patients 18 to 65 years of age with low back pain of mechanical origin. (mix/not specified)	• Chiropractic care: at the discretion of the chiropractor for a maximum of 10 treatments over one year. The treatments were intended to be concentrated within the first 3 months (n = 384). • Physiotherapy care: within hospital outpatient clinics (n = 357).	• Total: 3 years • Relevant for this review: 1 month (week 6), 1 year	- Functional status (Oswestry 0–100) - Global improvement (Number of patients partially or complete relieved)
- Petersen 2011[53] - Denmark, Primary care specialist center in Copenhagen	Patients of 18 and 60 years of age suffering from LBP with or without leg pain since more than 6 weeks. (sub-acute/chronic)	• Chiropractic care: all type of manual technique including SMT and myofascial trigger-point massage at the discretion of the chiropractor for a maximum of 15 treatments in a 12 weeks period. Mobilizing exercises, alternating lumbar flexion/extension movements, and stretching, were allowed (n = 175). • Physical therapy care: according to the McKenzie treatment protocols. An educational booklet about self-care or a “lumbar roll” for the seated posture were sometimes provided to the patient (n = 175).	• Total: 12 months (post-treatment completion) • Relevant for this review: 3 months (treatment completion (12 week)), 12 months (post-treatment completion)	- Functional status (Roland-Morris 0–23) - Back and leg pain (0–60 scale from 6 VAS (actual, worst, average)) - Health related quality of life (SF-36 general health perception and mental health scales (0–100)) - Global improvement (Number of patients scoring completely cured_ or much improved on a 6-point Likert scale (much worse, worsened, no change, improved, much improved, completely cured))

HMO: Health Maintenance Organization; SMT: Spinal Manipulative Therapy; VAS: Visual Analog Scale

* Results for the one-year follow-ups were only provided graphically and could not be used for this review

** Precisions regarding the chiropractic care modalities obtained from communication with the study authors

#### Risk of bias within studies

The methodological quality of the scientifically admissible studies is presented in Table 2. Most studies (5/6) had a low risk of bias with the exception of one which had a high or unclear risk of bias for the majority of the evaluated item (12/13) [52]. Among the low risk of bias studies, one publication provided an unclear description of the randomization [51] and another one did the same for the allocation concealment [43, 44]. As expected items related to the blinding of patients, care providers, and outcome assessors were at high risk of bias in every study due to the intervention and outcome considered in this review. Selective outcome reporting was suspected in one study reporting an unusual primary outcome [53]. Co-interventions use was different between groups in one study [45, 46] and unclear in another one [43, 44]. Compliance was different between the two treatments group in one study [53] and could not be clearly evaluated in two studies [43, 44, 51]. The possibility of an additional bias was suspected in one study that restricted the number of treatment sessions in only one of its treatment groups [43, 44].

**Table 2:** Risk of bias of the included clinical effectiveness studies of chiropractic care for non-specific low back pain.

	Random sequence generation	Allocation concealment	Blinding of participants	Blinding of personnel /care providers	Blinding of outcomes assessors	Incomplete outcome data	Selective outcome reporting	Group similarity at baseline	Co-interventions	Compliance	Intention-to-treat-analysis	Timing of outcome assessments	Other bias	Overall risk of bias
Bronfort 2011[50]	Low	Low	High	High	High	Low	Low	Low	Low	Low	Low	Low	Low	Low
Cherkin 1998 [51]	Unclear	Low	High	High	High	Low	Low	Low	Low	Unclear	Low	Low	Low	Low
Herzog 1991 [52]	Unclear	Unclear	High	High	High	High	Unclear	High	Low	Unclear	High	High	High	High
Hurwitz 2002 [45, 46]	Low	Low	High	High	High	Low	Low	Low	High	Low	Low	Low	Low	Low
Meade 1990 [43, 44]	Low	Unclear	High	High	High	Low	Low	Low	Unclear	Unclear	Low	Low	High	Low
Petersen 2011[53]	Low	Low	High	High	High	Low	High	Low	Low	High	Low	Low	Low	Low

### Summary of evidence

#### Chiropractic care vs exercise therapy care

Bronfort et al. [50] compared chiropractic care to exercise therapy in the United States for LBP subjects of at least six weeks duration. The extracted results are presented in Table 3. Outcomes of pain, functional status and global improvement assessed at one, three and 12 months revealed no significant difference between the two provider groups. The responder analysis (proportion of patients with at least 50% and 75% improvement) was coherent with the averaged results and no significant difference was found between the two types of provider for pain, functional status and global improvement assessed at three and 12 months.

**Table 3:** Chiropractic care versus Exercise therapist care.

			Chiropractic				Exercise therapist				Overall	
Study	Outcome	Time	Mean	SD	N	% of change from baseline	Mean	SD	N	% of change from baseline	Standardized mean difference (95% CI)	P-value
Bronfort 2011 (sub-acute/chronic)	Pain	1 month	3.9	1.8	100	-27.8	3.7	1.8	95	-27.5	0.11 (-0.17, 0.39)	0.44
		3 month	2.9	1.9	99	-46.3	2.6	2.1	93	-49.0	0.15 (-0.13, 0.43)	0.30
		12 month	3.3	2.1	81	-38.9	2.8	2.3	82	-45.1	0.23 (-0.08, 0.53)	0.15
	Functional status	1 month	5.9	4.9	100	-32.2	5.9	4.4	94	-29.8	0.00 (-0.28, 0.28)	1.00
		3 month	4.9	5.0	99	-43.7	3.9	4.6	92	-53.6	0.21 (-0.08, 0.49)	0.15
		12 month	5.1	4.9	81	-41.4	3.8	4.7	82	-54.8	0.27 (-0.04, 0.58)	0.09
	Health related quality of life (SF-36 Physical scale)	1 month	46.2	7.1	100	7.9	47.2	8.0	94	8.0	-0.13 (-0.41, 0.15)	0.36
		3 month	48.0	7.7	99	12.1	49.7	7.8	92	13.7	-0.22 (-0.50, 0.07)	0.13
		12 month	48.4	8.0	81	13.1	50.4	7.2	82	15.3	-0.26 (-0.57, 0.05)	0.10
	Health related quality of life (SF-36 Mental scale)	1 month	56.0	6.7	100	1.6	53.9	9.1	94	0.4	0.26 (-0.02, 0.55)	0.07
		3 month	57.2	5.3	99	3.8	55.2	7.8	92	2.8	0.30 (0.02, 0.59)	**0.04**
		12 month	55.2	7.5	81	0.2	53.9	8.6	82	0.4	0.16 (-0.15, 0.47)	0.31
	Global improuvement	1 month	3.5	1.2	100	Not available	3.8	1.0	94	Not available	-0.27 (-0.55, 0.01)	0.06
		3 month	2.9	1.4	99	Not available	2.7	1.3	92	Not available	0.15 (-0.14, 0.43)	0.31
		12 month	3.3	1.6	81	Not available	3.1	1.6	82	Not available	0.12 (-0.18, 0.43)	0.43

Health related quality of life was assessed at one, three and 12 months using the physical and mental health subscales of the SF-36. The only statistically significant difference favour chiropractic care for the mental subscale at three months follow-up.

The rate of adverse effects did not differ significantly between the two treatment groups (Table 4). These were rare (2% and 6% for chiropractic and exercise therapy care respectively), transient in nature, and necessitated minimal or no change in activity level.

**Table 4:** Adverse events.

Study	Adverse events	N	Adverse events	N	Risk Ratio (95% CI)	P-value
Bronfort 2011 (sub-acute/chronic)	**Chiropractic**		**Exercise therapist**			
	2	100	6	100	0.33 (0.07, 1.61)	0.17
Cherkin 1998 (mix/not specified)	**Chiropractic**		**Physical therapy**			
	0	122	0	133	Not estimable	Not applicable
Hurwitz 2002 (mix/not specified)	**Chiropractic**		**Medical**			
	0	169	0	170	Not estimable	Not applicable

#### Chiropractic care vs physical therapy care

**Fig 2:**
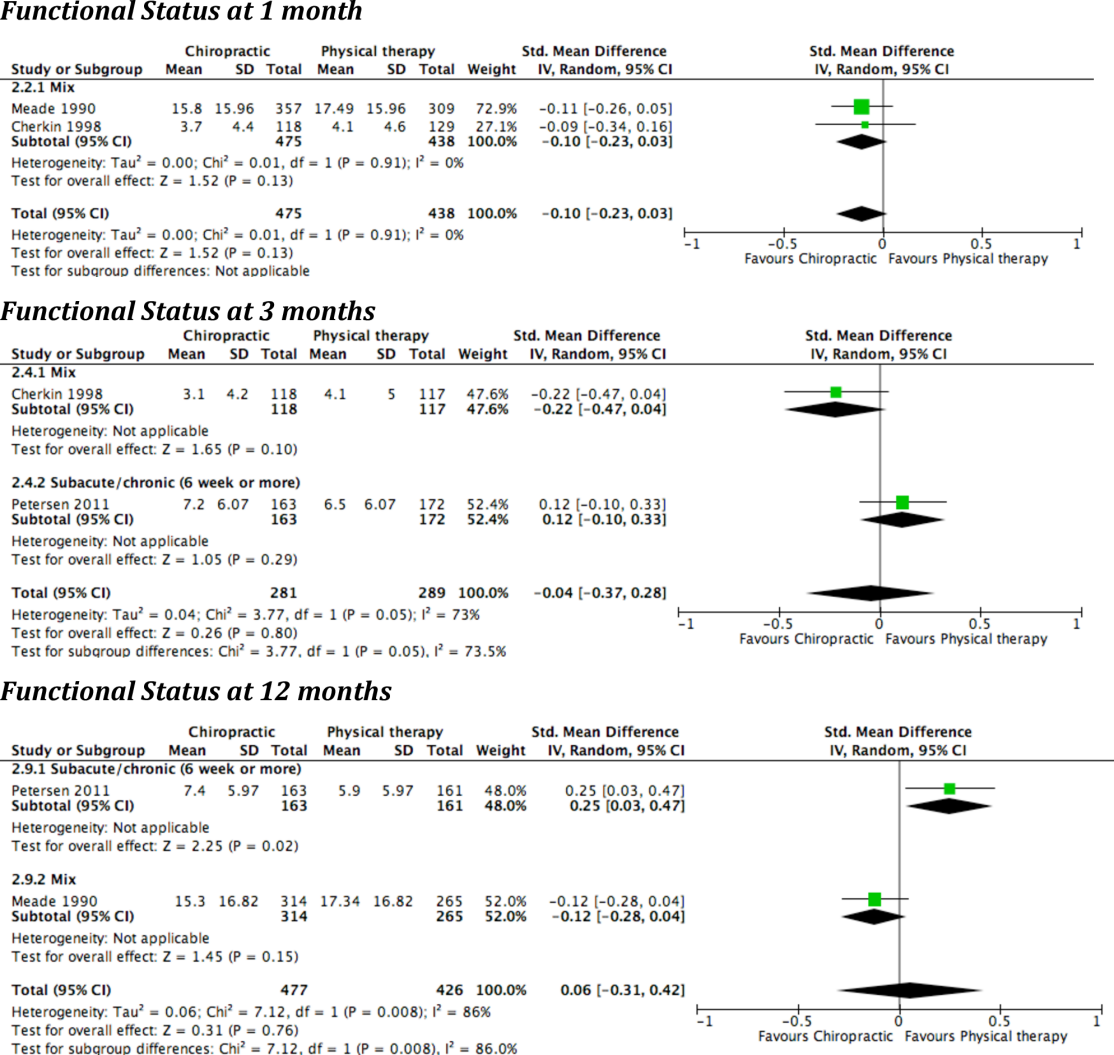
Forest plots of comparison: Chiropractic care versus Physical therapy care.

Three studies compared chiropractic to physical therapy care; they were conducted in the United-States [51], United Kingdom [43, 44] and Denmark [53]. Two included a mix of LBP duration [43, 44, 51] and one included subjects with symptom onset of six weeks or more [53]. The extracted results and pooled effect are presented in Table 5 and Fig 2.

**Table 5:** Chiropractic care versus Physical therapy care.

			Chiropractic				Physical therapy				Overall	
Outcome	Time	Study	Mean	SD	N	% of change from baseline	Mean	SD	N	% of change from baseline	Standardized mean difference (95% CI)	P-value
Pain reduction from baseline	3 month	Petersen 2011 (sub-acute/chronic)	13.8	13.0	163	-47.4	15.4	13.4	172	-51.2	-0.12 (-0.33, 0.09)	0.27
	12 month	Petersen 2011 (sub-acute/chronic)	12.2	13.7	163	-42.1	15.0	13.6	161	-50.0	-0.20 (-0.42, 0.01)	0.07
Functional status	1 month	Meade 1990 (mix/not specified)	15.8	16.0	357	-47.0	17.5	16.0	309	-38.6	-0.10 (-0.23, 0.03)	0.13
		Cherkin 1998 (mix/not specified)	3.7	4.4	118	-69.4	4.1	4.6	129	-66.4		
	3 month	Cherkin 1998 (mix/not specified)	3.1	4.2	118	-74.4	4.1	5.0	117	-66.4	-0.04 (-0.37, 0.28)	0.80
		Petersen 2011 (sub-acute/chronic)	7.2	6.1	163	-44.6	6.5	6.1	172	-50.0		
	12 month	Petersen 2011 (sub-acute/chronic)	7.4	6.0	163	-43.1	5.9	6.0	161	-54.6	0.06 (-0.31, 0.42)	0.76
		Meade 1990 (mix/not specified)	15.3	16.8	314	-48.7	17.3	16.8	265	-39.2		
Health related quality of life (General)	3 month	Petersen 2011 (sub-acute/chronic)	69.5	19.6	163	6.9	72.1	19.6	172	7.6	-0.13 (-0.35, 0.08)	0.23
	12 month	Petersen 2011 (sub-acute/chronic)	65.3	23.0	163	0.5	69.5	23.0	161	3.7	-0.18 (-0.40, 0.04)	0.10
Health related quality of life (Mental)	3 month	Petersen 2011 (sub-acute/chronic)	74.2	20.2	163	14.2	74.2	20.2	172	14.2	0.00 (-0.21, 0.21)	0.99
	12 month	Petersen 2011 (sub-acute/chronic)	73.8	20.4	163	13.5	76.2	20.4	161	17.2	-0.12 (-0.34, 0.10)	0.29
			**Events**		**N**		**Events**		**N**		**Risk Ratio (95% CI)**	**P-value**
Global improuvement	1 month	Meade 1990 (mix/not specified)	312		360		245		317		1.12 (1.04, 1.21)	**0.002**
	3 month	Petersen 2011 (sub-acute/chronic)	53		153		81		169		0.72 (0.55, 0.95)	**0.02**

Pain was assessed at three and 12 months by only one study. There was no significant difference between the two treatment groups.

Functional status was assessed at 1 month (n = 2), 3 months (n = 2) and 12 months (n = 2). None of the individual studies or the pooled effects showed significant between group differences. The only exception was the Danish study [53], in which a small statistically significant effect in favour of physical therapy care at the 12 months follow-up was reported. The responder analysis (number of patients with an absolute Roland Morris score below 5 points or at least 5 points reduction) of that study revealed no significant between group difference for functional status at three and 12 months. This study was the only one to report functional status at 12 months for subacute/chronic subpopulation. Nevertheless, the pooled effect, including results of the Meade study [43, 44] from a mixed subpopulation, did not reveal significant between group differences. The heterogeneity analysis of the pooled three and 12 months outcomes showed substantial heterogeneity. Potential sources were: different settings (USA, UK and Denmark), different subpopulations in terms of symptom duration, and different physical therapy approaches (McKenzie method) and usual hospital outpatient clinic.

Global improvement was assessed at one and three months in one study. At one month, Meade et al. [43, 44] reported a statistically significant advantage in favour of chiropractic care, while at three months Petersen et al. [53] reported a statistically significant advantage in favour of physical therapy care.

Health related quality of life was only reported by Petersen et al. [53] at three and 12 months using the general health perception and mental health subscales of the SF-36. None of the comparisons significantly statistically favoured one type of care.

Return to work was also reported in the Danish study [53]. Since the two treatment groups were significantly different regarding their number of participants off work at baseline, we chose not to extract data for that particular outcome.

Adverse events were only reported in the Cherkin study [51], and no serious adverse effects were recorded in either of the treatment groups (Table 4).

#### Chiropractic care vs medical care

Only one study compared chiropractic care to medical care. The study was conducted by Hurwitz et al. in the United States and included subjects with a mixed symptom duration of LBP [45, 46]. Extracted results for this study are presented into Table 6.

**Table 6:** Chiropractic care versus Medical care.

			Chiropractic			Medical			Overall	
Study	Outcome	Time	Mean	SD	N	Mean	SD	N	Standardized mean difference (95% CI)	P-value
Hurwitz 2002 (mix/not specified)	Pain	1 month	3.6	2.58	169	3.86	2.58	169	-0.10 (-0.31, 0.11)	0.36
		12 month	3.1	2.54	153	3.31	2.54	153	-0.08 (-0.31, 0.14)	0.47
	Functional status	1 month	7.5	5.91	169	7.87	5.91	169	-0.06 (-0.28, 0.15)	0.57
		12 month	7.05	6.02	153	7.00	6.02	153	0.01 (-0.22, 0.23)	0.94

Pain and functional status were assessed at one and 12 months. There was no significant difference between the two treatment groups.

Global improvement, health related quality of life, and return to work were not assessed. No serious adverse events were recorded in neither of the treatment group (Table 4).

Overall, the pooled results did not show significant differences in effectiveness between chiropractic care and the other type of standard care studied. When reported, no serious adverse events were recorded in any of the chiropractic, exercise therapy [50], physical therapy [51] or medical [45, 46] treatment groups (Table 4).

### Economic evaluation

#### Study selection

**Fig 3:**
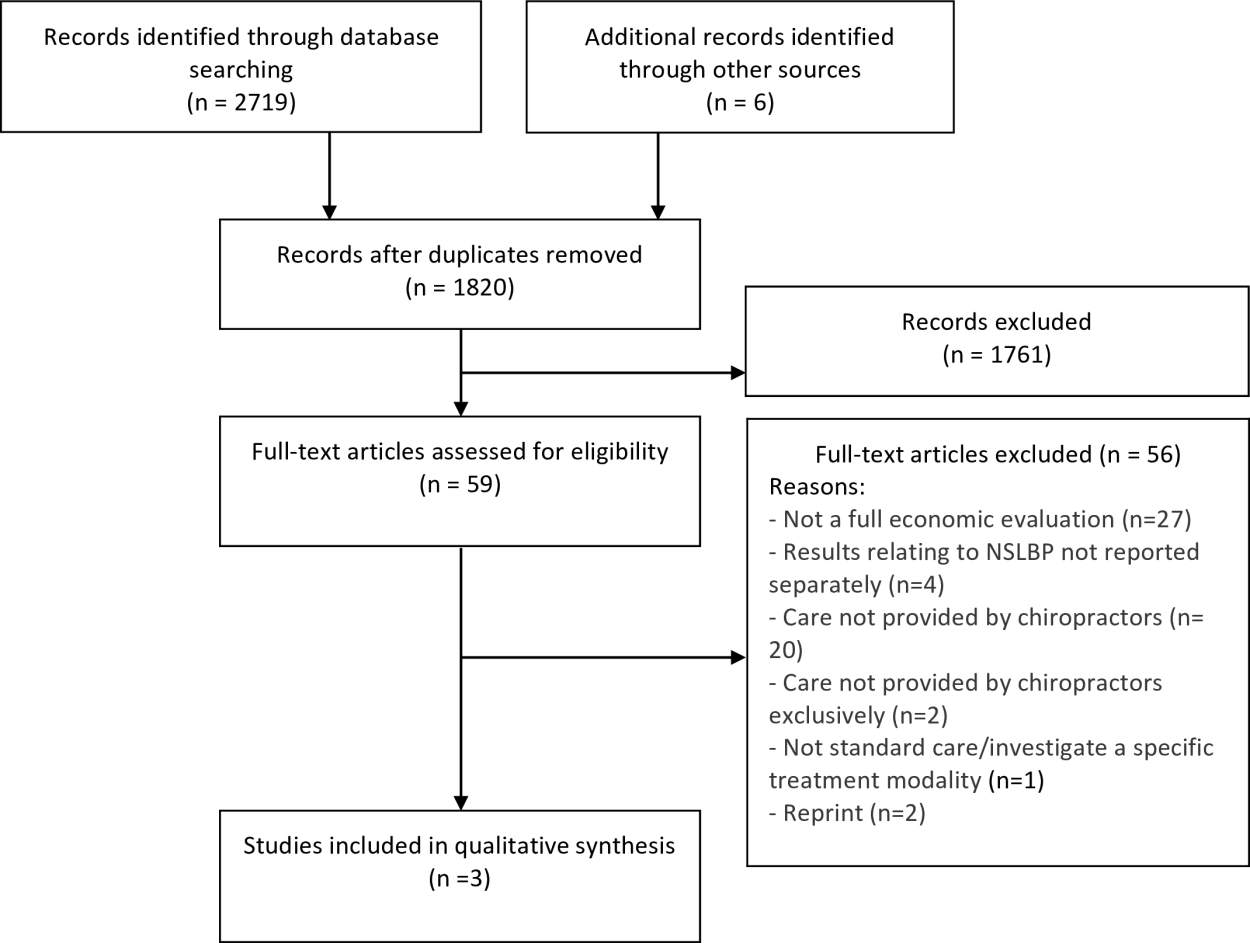
Flow diagram for the selection of economic evaluations.

Our search yielded 2,725 citations. We removed 905 duplicates and screened 1,820 articles for eligibility (Fig 3). After screening, 1,761 articles did not meet our selection criteria. Fifty-nine articles were assessed for eligibility and only three articles were scientifically admissible. The main reasons for exclusion of the full papers were: partial economic evaluations or lack of any cost evaluation.

#### Study characteristics

The characteristics of these included studies are presented in Table 7. All three included studies were conducted in the United-States, included between 417 and 2780 participants, and involved follow-up periods ranging from 1 to 3.7 years. The types of economic evaluation performed were: cost-effectiveness analysis (n = 1) [54], cost-benefits analysis (n = 1) [55], and cost-minimizations analysis conducted along with a randomized clinical trial (n = 1) [56]. All three included studies compared chiropractic care to combinations of medical care. The quality assessment of the included studies is reported in Table 8.

**Table 7:** Characteristics, key findings of economic evaluations of Chiropractic care for non-specific low back pain.

First Author, Year, Country, Type of economic evaluation	Participants, Indication and Setting	Comparative Treatments	Perspective, Time Horizon, Currency Price (Year)	Included Costs, Health Effects	Mean Health effect, Mean Costs (2015 USD)*	Incremental Cost-effectiveness, Incremental net-benefit
Butler, 2010 [55]USCost-benefit analysis/prospective cohort	417 adults workersOccupational LBPFive employers with establishments in 37 states recruited for The Arizona State University Healthy Back Study	Medical doctors or osteopaths (MD/DO)(n = 20)MD/DO combined with Physical therapy (MDPt)(n = 144)Chiropractors (DC)(n = 15)DC and MD/DOs (n = 105)Surgeons and MDs in emergency departments (Sx/ED)(n = 133)	Employer (society)3.7 years2002 US dollars	Costs:	Adjusted Health effects:	Adjusted Net-benefits:
				Offices visits Consultations Physical medicine X-rays Medication	MD/DO: 147,113$ MDPt: 139109$ DC: 142,053$ DC and MD/DO: 115,301$ Sur/ED: 87,661$	MD/DO: 135,824$ MDPt: 130,064$ DC: 132,989$ DC and MD/DO: 104,025$ Sur/ED: 60,807$
				Health effects:	Adjusted costs:	
				- Saving in work loss day in comparison of worker not returning to work (days x wage)	MD/DO: 11,289$ MDPt: 9046$ DC: 9065$ DC and MD/DO: 11277$ Sx/ED: 26,854$	
Haas, 2005 [54]USCost-effectiveness analysis/ prospective cohort	2780 ambulatory adultsLBP of mechanical origin (acute and chronic (7weeks)51 chiropractic clinics and 14 general practice community clinics in Oregon and Washington	Chiropractors (n = 1328 acute and 527 chronic)Medical doctors (n = 615 acute and 310 chronic)	Healthcare system (Medicare)**12 monthsConstant 1995 US dollars	Costs:	Adjusted mean differences DC-MD (SD)	Cost-effectiveness ratio
				Office visit Radiograph Medication Advanced imaging (imputed) Surgical consultation (imputed) Physical therapists referrals (imputed)	*Acute patients*	*Acute patients*
					Pain: 3.6 (1.3)Disability: 2.7 (1.1)Physical health: 9.2(2.5)Mental health: 5.4 (2.5)Satisfaction: 14.0 (3.1)	Pain: 17.6$Disability: 23.6$Physical health: 6.9$Mental health: 11.7$Satisfaction: 4.5$
					Total cost: 63$ (69$)	
				Health effects:	*Chronic patients*	*Chronic patients*
				Pain (100mm VAS)Functional status (Oswestry 100 point scale)Physical health (SF-12)Mental health (SF-12) Satisfaction (100 point scale)	Pain: 7.3 (2.1)Disability: 5.4 (1.7)Physical health: 3.0 (3.6)Mental health: 1.2 (3.7)Satisfaction: 18.1 (4.9)	Pain: 0.1$Disability: 0.1$Physical health: 0.3$Mental health: 1.0$Satisfaction: 0.0$
					Total cost: 1.5$ (117$)	
Kominski, 2005 [56]USCost minimisation analysis/ randomized controlled trial	681 adults members of various HMOsLBP (with or without leg symptoms)Large medical group practice with 3 sites in Southern California	Medical care (MD)(n = 162)Medical care with physical therapy (MDPt)(n = 167)Chiropractic care (DC)(n = 162)Chiropractic care with physical modalities (DCPm)(n = 163)	Healthcare provider group **18 months1998 US dollars	Costs (charged):	Mean cost (SD):	Not reported
				Office visits Diagnostic services Therapeutic services	MD: 647$ (1755)MDPt: 1070$ (1454)DC: 769$ (1166)DCPm: 790$ (765)	
				Health effects:	Mean Health effect:	
				Pain IntensityFunctional status (Roland-Morris)	No significant differences between groups	

* Cost of the original study were converted to 2015 US dollar using a web-based tool based PPP and GDPD values from the IMF[47]

** Precision obtained directly from the original author

**Table 8:** Quality assessment of economic evaluation of chiropractic cares for non-specific low back pain.

Author (year)		Butler (2010)	Haas (2005)	Kominski (2005)
**Study design**				
**1**	**Was a well-defined question posed in answerable form?**	Yes	Not Clear	Yes
1.1	Did the study examine both costs and effect of the service (s) or programme (s)?	Yes	Yes	No
1.2	Did the study involve a comparison of alternatives?	Yes	Yes	Yes
1.3	Was a viewpoint for the analysis stated and was the study placed in any particular decision-making context?	Yes	No	No
**2**	**Was a comprehensive description of the competing alternative given? (that is, can you tell who did what to whom, where, and how often?)**	Yes	Yes	Yes
2.1	Were any relevant alternatives omitted?	No	No	No
2.2	Was (Should) a do-nothing alternative (be) considered?	Not appropriate	No	No
**3**	**Was the effectiveness of the programme or services established?**	Yes	Yes	Yes
3.1	Was this done through a randomized, controlled clinical trial? If so, did the trial protocol reflect what would happen in regular practice?	No	No	Yes
3.2	Were effectiveness data collected and summarized through a systematic review of studies? If so, were the search strategy and rules for inclusion or exclusion outlined?	No	No	No
3.3	Were observational data or assumptions used to establish effectiveness? If so, what were the potential biases in the results?	Yes	Yes	No
**4**	**Were all the important and relevant costs and consequences for each alternative identified?**	Yes	Yes	No
4.1	Was the range wide enough for the research question at hand?	Yes	Yes	Yes
4.2	Did it cover all relevant viewpoints? (Possible viewpoints include the community or social viewpoints, and those of patients and third-party payers. Other viewpoints may also be relevant depending upon the particular analysis.)	Yes	Yes	No
4.3	Were capital costs, as well as operating costs, included?	No	No	No
**5**	**Were costs and consequence measured accurately in appropriate physical units (e.g. hours of nursing time, number of physician visits, lost work days, gained life-years)?**	Yes	Yes	No
5.1	Were the sources of resources utilisation described and justified?	Yes	Yes	No
5.2	Were any of the identified items omitted from measurement? If so, does this mean that they carried no weight in the subsequent analysis?	No/No	Yes/No	Yes/Yes
5.3	Were there any special circumstances (e.g. joint use of resources) that made measurement difficult? Were these circumstances handled appropriately?	Yes/Yes	Yes/Yes	Not clear
**6**	**Were costs and consequences valued credibly?**	Yes	Yes	No
6.1	Were the sources of all values clearly identified? (Possible sources include market values, patient or client preferences and views, policymakers’ views and health professionals’ judgments.)	Yes	Yes	Yes
6.2	Were market values employed for changes involving resources gained or depleted?	Yes	Yes	Yes
6.3	Were market values were absent (for example, volunteer labour), or market values did not reflect actual values (such as clinic space donated at a reduce rate), were adjustments made to approximate market values?	Yes	Yes	No
6.4	Was the valuation of consequences appropriate for the question posed (that is, has the appropriate type of analysis (CEA, CUA, CBA) been selected)?	Yes	Yes	No
**7**	**Were costs and consequences adjusted for differential timing?**	Not appropriate	Not appropriate	Not appropriate
7.1	Were costs and consequences that occur in the future “discounted” to their present values?	Not appropriate	Not appropriate	Not appropriate
7.2	Was any justification given for the discount rate(s) used?	Not appropriate	Not appropriate	Not appropriate
**8**	**Was an incremental analysis of costs and consequences of alternative performed?**	Yes	Yes	No
8.1	Were the additional (incremental) costs generated by one alternative over another compared with the additional effects, benefits, or utilities generated?	Yes	Yes	No
**9**	**Was allowance made for uncertainty in the estimation of costs and consequences?**	No	Yes	Yes
9.1	If patient level data on cost or consequence were available, were appropriate statistical analysis performed?	Yes	Yes	Yes
9.2	If a sensitivity analysis was employed, was justification provided for the ranges or distributions of values (for key study parameters), and the form of sensitivity analysis used?	No	No	No
9.3	Were the conclusions of the study sensitive to the uncertainty in the results, as quantified by the statistical and/or sensitivity analysis?	Yes	Yes	Yes
**10**	**Did the presentation and discussion of the study results include all issues of concern to user?**	No	Yes	Yes
10.1	Were the conclusions of the analysis based on some overall index or ratio of costs to consequences (e.g. costs effectiveness ratio)? If so, was the index interpreted intelligently or in a mechanistic fashion?	Yes	Yes	No
10.2	Were the results compared with those of other who have investigated the same or similar questions? If so, were allowances made for potential differences in study methodology?	No	Yes	Yes
10.3	Did the study discuss the generalizability of the results to other settings and patient/client groups?	No	Yes	Yes
10.4	Did the study allude to, or take account of, other important factors in the choice or decision under consideration (e.g., distribution of costs and consequences, or relevant ethical issues)?	No	Yes	Yes
10.5	Did the study discuss issues of implementation, such as the feasibility of adopting the preferred programme given existing financial or other constraints, and whether any freed resources could be redeployed to other worthwhile programmes?	No	Yes	No
	**Overall quality assessment of the study**	Low	High	Medium

#### Summary of evidence and quality assessment

Butler et al. [55] conducted a cost-benefit study in the US from the perspective of the employer as a proxy of the societal perspective. The authors used data from a prospective cohort study as an exemplar to present a method for adjusting rehabilitation costs and benefits for health capital. The cohort consisted of patients with occupational low back pain from five employers across 37 federal states. Chiropractic care (n = 15 patients) was compared to different types of providers: medical doctors (n = 20 patients), medical doctors and physical therapists (MDPt, n = 144 patients), chiropractors and medical doctors (n = 105 patients), and surgeons or emergency physicians (n = 133 patients). Direct costs (offices visits, consultations, physical medicine, radiographs, medication) and indirect costs (saving from work loss day) were considered. Costs related to the household sector were not included. Based on the nearly identical adjusted net benefits for physician only care (135,824$), physician plus physical therapy care (130,064$), and chiropractic care (132,989$), the three types of care seemed equivalent. Net benefits of care were lower for combined physician/chiropractic care (104,025$), and lowest for all other forms of care (104,025$). Precision of the estimates and sensitivity analysis were not reported. Butler et al. presented the analysis as an exemplar and therefore the sample size in the chiropractic group was low. With only 15 patients receiving chiropractic care, the parametric multivariate analyses conducted by the author is likely underpowered and their conclusions therefore not robust. Based on this, we rated the study quality as low despite the high quality of their methodology.

Haas et al. [54] conducted a cost effectiveness study from the perspective of the healthcare system (Medicare) based on a prospective cohort study. They recruited participants from 51 chiropractic clinics (n = 60 providers and n = 1855 patients) and 14 general practice community clinics (n = 111 medical providers and n = 925 patients) in Oregon and Washington, US. The effectiveness measures were based on patient- reported outcomes including pain, functional status, treatment satisfaction, and physical and mental health. The following direct costs were considered: office visits, radiographs and medication. The costs related to advance imaging [57], surgical [58] and physical therapy consultations [51] were imputed from previous published studies. The services provided were assigned Current Procedural Terminology (CPT) codes that were converted to Medicare relative value units, and a conversion factor was applied to estimate the Medicare payment. The adjusted mean differences (AMDs) of total cost at 12 months revealed that chiropractic care was slightly more expensive than medical care for acute (63$, P = 0.352) and chronic patients (1.5$, p = 0.993), but the differences were not significant. The AMDs for pain and functional status at 12 months significantly advantaged chiropractic care, but were not clinically important (AMDs < 10, P < 0.01). The ICER ranged from 4.5$ to 23.6$ per unit of change of the considered outcomes for the acute patients and from 0.0$ to 4.5$ for the chronic patients. Formal willingness to pay and sensitivity analysis were not reported. Given the very small ICER for the chronic patients, chiropractic care appears relatively cost effective. The interpretation is less clear for acute patients, but chiropractic and medical care appear to perform comparably. Our overall assessment of the study lead us to rate this study as high quality, because it considered all the costs and outcomes relevant to the perspective of the analysis.

Kominski et al. [56] conducted a cost-minimization analysis alongside the randomized controlled trial reported by Hurwitz[45, 46] from the perspective of the healthcare provider group. The authors recruited Health Maintenance Organization (HMO) patients from three large medical group practices in Southern California, US. Chiropractic care (n = 162) was compared to medical care (n = 162), medical care combined with physical therapy (n = 167) and chiropractic care combined with physical modalities (n = 163). The medical group received capitated payments from the insurer. The costs in this study were imputed using CPT codes and a Medicare fee schedule. The following direct costs were considered: offices visits, diagnostic and therapeutic services. The mean total cost for chiropractic care (769$) was significantly higher than medical care (647$) without producing significantly better clinical outcomes. The addition of physical therapy (1070$) to medical care and physical modalities to chiropractic care (790$) also generated greater costs for similar clinical outcomes. Sensitivity analyses were not reported. The study quality was rated as medium, because the healthcare provider group used a remuneration system that likely differed from the Medicare one. As a consequence, the method used for cost imputation might not reflect the actual cost of the healthcare provider group. Additionally, medication costs were not considered. Inclusion of these costs might considerably influence the overall care cost, and in particular the medical care costs, and thereby the study conclusions.

In summary, according to the Slavin’s best-evidence synthesis approach, the level of evidence of the economic findings relating to chiropractic compared to medical care is mixed as the three included studies reported inconsistent conclusions regarding chiropractic care.

## Discussion

### Summary of findings

We identified six clinical effectiveness RCTs and three full economic evaluations. Overall, individual studies showed similar effects of chiropractic care compared to exercise therapy, physical therapy or medical care for the treatment of low back pain regardless of type of outcome, and the risk of bias was low in five out of six included studies. Similarly, the pooled results revealed no significant difference in effectiveness between providers groups. No serious adverse events were reported for any treatment approaches. The three full economic evaluation studies comparing chiropractic care to medical care were of low to high methodological quality. These studies provided conflicting results regarding cost-effectiveness, and the resulting level of evidence is mixed.

Unfortunately, very few studies met our inclusion criteria and all our effect estimates regarding primary and secondary outcomes came from one or two studies. Additionally, two of our pooled estimates included a considerable level of heterogeneity [59]. Therefore, future high quality studies are likely to influence effect estimates. In three of the included clinical effectiveness studies, we found individual outcomes that were not in concordance with our overall findings. Most notable was the study by Petersen et al., in which physical therapy care resulted in better global improvement at three months and higher functional status at 12 months, compared to chiropractic care in a population of subacute/chronic LBP [53]. When results were pooled with those from the other available studies for functional status at three [51] and 12 months [43, 44], the heterogeneity was considerable [59]. The inconsistent results can be explained by differences in settings (studies conducted in Denmark, UK or US); subpopulations (subacute/chronic vs. mixed symptom duration), and physical therapy approaches (McKenzie method vs. non-specified physical therapy). We found one study, which favoured chiropractic care over physical therapy care in a mixed duration LBP population regarding global improvement at one month [43, 44]. Finally, during our data extraction for the pooled effect estimates we found one outcome (the mental subscale of the SF-36 at the three months follow-up) that favoured chiropractic care over exercise therapy care in the study by Bronfort et al. [50]. The authors did not report this difference because they performed a multivariate analysis (instead of a final score comparison), which led them to report a non-significant difference.

The existing literature is currently too limited to allow for a synthesis of economic evaluations robust enough to offer guidance to decision makers, referring healthcare provider and patients about the cost-effectiveness of chiropractic compared to other types of care. Two out of three included economic evaluations had serious methodological limitations due to low sample size and omission of direct medical costs that could potentially impact the conclusions. Further, it is difficult to appreciate the robustness of the conclusions of the economic evaluations considering that sensitivity analyses were not reported.

### Consistency of findings with other studies and reviews

Our results regarding the effectiveness of chiropractic care are consistent with previous reviews comparing chiropractors to other provider groups [21, 22] and SMT to other treatment modalities [24–26]. All have concluded that there is no clear superiority for any provider group or modality. Regarding economic evaluations, older reports [60, 61] have concluded that chiropractic care is highly cost-effective because of the relatively low consultation fee and the limited use of advanced diagnostic imaging. However, more recent rigorous systematic reviews of partial economic evaluations have failed to show an economic advantage of one type of care over another [21, 22]. This is consistent with the findings from our review based on a limited number of full economic evaluations.

### Strengths and limitations of these reviews

To our knowledge this is the first synthesis that exclusively considers pragmatic trials of chiropractic care without including fastidious trials of SMT. A comprehensive search was conducted to identify all relevant studies and a rigorous methodology was applied. The extraction process was performed in accordance with current guidelines and an experienced health economist supported the economic evaluations. Limiting our search to the past 25 years enabled us to select publications that were compatible with contemporary healthcare delivery. By only considering full economic evaluations and trials at a low risk of bias, we aimed to derive conclusions from more robust evidence.

Our review has limitations. First, we did not search the grey literature for clinical effectiveness studies. McAuley et al. showed that the inclusion of results from the grey literature tend to decrease effectiveness estimates in meta-analyses because the unpublished studies tend to report smaller treatment effects [62]. Second, critical appraisal requires scientific judgment that may vary among reviewers. This potential bias was minimized by training reviewers to use a standardized critical appraisal tool and using a consensus process among reviewers to reach decisions regarding scientific admissibility. Most of the original between-group differences and pooled estimates in our meta-analysis did not favour a specific provider group, and we believe it is unlikely that the inclusion of unpublished grey literature would change our conclusions. Third, the low number of clinical trials prevents us from conducting a meaningful investigation for publication bias. Fourth, the majority of the included clinical effectiveness studies (three out of five) and all three economic evaluations were conducted in the United States. Caution should therefore be used when generalizing our findings to other settings or jurisdictions. With respect to economic evaluations in particular, local healthcare systems and insurance plans may have a higher impact on cost than the type of healthcare provider [55].

### Recommendations for future researches

Our search retrieved only a limited number of pragmatic clinical trials comparing chiropractic care to other types of care and only one high quality, full economic evaluation. We suggest therefore that future high quality pragmatic trials be conducted in parallel with full economic evaluations considering all relevant direct and indirect costs. The perspective of the economic evaluation should be clearly specified since it bears influence on the relevance of the health effects and cost considered. In addition, future studies should consider the impact of chiropractic care on return to work and include loss of income and wage compensation, a major component of indirect cost relevant to patients, the employers and society. Small differences in work absenteeism (indirect cost) have previously shown to have considerable impact on cost and the potential to reverse conclusions based on direct costs only [63, 64]. To facilitate the interpretation and decision-making process about types of care, we also suggest that future economic evaluations conduct willingness to pay and sensitivity analyses. Due to the large numbers of fastidious trials investigating the efficacy of SMT of LBP, researchers have previously called for a moratorium on future RCTs [26, 65]. However, we believe that additional pragmatic, practice-based studies will help clarify whether chiropractic care is truly equivalent to other types of care in terms of effectiveness and cost. It is likely that, on average, there is not much difference between the different alternatives; however there may be heterogeneous treatment effects, i.e. some treatments that are cost-effective for some and not for others. Future studies could help elucidate this issue.

## Conclusion

Moderate evidence suggests that chiropractic care for LBP appears to be equally effective as physical therapy. Limited evidence suggests the same conclusion when chiropractic care is compared to exercise therapy and medical care although no firm conclusion can be reached at this time. No serious adverse events were reported for any type of care. Our review was also unable to clarify whether chiropractic or medical care is more cost-effective. Given the limited available evidence, the decision to seek or to refer patients for chiropractic care should be based on patient preference and values. Future studies are likely to have an important impact on our estimates as these were based on only a few admissible studies.

## Supporting Information

S1 Table PRISMA Checklist. (DOC)

## Abbreviations

AMD: Adjusted Mean Difference
BMJ: British Medical Journal
CADTH: Canadian Agency for Drugs and Technologies in Health
CIHI: Canadian Institute for Health Information
CIHR: Canadian Institute of Health Research
CPGs: Clinical Practice Guidelines
CPT: Current Procedural Terminology
DC: Doctor of Chiropractic
DCPm: Chiropractic care with physical modalities
GDPD: Gross Domestic Product Deflator
HMO: Health Maintenance Organization
ICL: Index to Chiropractic Literature
LBP: Low Back Pain
MD: Medical Doctor
MD/DO: Medical Doctor or Doctor of Osteopathy
MDPt: Medical Doctor and Physical therapy
NICE: National Institute for Health and Care Excellence
PPP: Purchasing Power Parities
QALYs: Quality Adjusted Life Years
RCTs: Randomized controlled trial
SD: Standard Deviation
SMT: Spinal Manipulative Therapy
Sx/ED: Surgeons and Emergency Department
US: United-States
USD: United-States Dollard
UK: United Kingdom
VAS: Visual Analogue Scale
